# Predicting the impact of climate change and land use change on the potential distribution of two economic forest trees in Northeastern China

**DOI:** 10.3389/fpls.2024.1407867

**Published:** 2024-07-12

**Authors:** Xiaokun Lin, Baoliang Chang, Yanqing Huang, Xin Jin

**Affiliations:** ^1^ Liaoning Institute of Forest Management, Dandong, China; ^2^ CAS Key Laboratory of Forest Ecology and Silviculture, Institute of Applied Ecology, Chinese Academy of Sciences, Shenyang, China; ^3^ Liaoning Shenyang Urban Ecosystem National Observation Research Station, Shenyang, China; ^4^ Shenyang Arboretum, Chinese Academy of Sciences, Shenyang, China

**Keywords:** *Aralia elata*, *Eleutherococcus senticosus*, environment variables, non-timber forest products, MaxEnt model, suitable habitat

## Abstract

Young shoots of *Aralia elata* and young leaves of *Eleutherococcus senticosus* are two major non-timber forest products in northeastern China. However, human activities and climate change have resulted in serious threats to the habitats of two trees, which greatly limits resource conservation and exploitation of economic forest trees. We used the MaxEnt model to predict the suitable habitats of the two economic trees and analyzed the dominant factors affecting their distribution. The results showed that the suitable habitat areas of *A. elata* and *E. senticosus* in the current period were 159950 km^2^ and 123449 km^2^, respectively, and the suitable habitats of both economic forest trees were located in the eastern part of the northeast region. Climate factors (Annual precipitation, Precipitation Seasonality) and land use factors are important variables influencing changes in suitable habitat for both trees. With the change of climate and land use in the future, the overall trend of suitable habitat for both economic forest trees shows a northward and then a southward migration. These results may provide assistance in developing strategies for resource conservation and sustainable use of *A. elata* and *E. senticosus*, and we suggest that stable and suitable habitats should be selected as areas for *in situ* conservation and breeding of the two economic forest trees.

## 1 Introduction

Forests have multiple values for human livelihoods, not least in terms of timber products (Ormbsby et al., 2021). In fact, people have an even longer history of obtaining food, medicinal and other plant-based products and their processed products from forests (Chamberlain and Smith-Hall, 2024). Non-timber forest products (NTFPs) are used for household livelihoods, economic income and culture, thereby contributing to human well-being (Nguyen et al., 2021). The main woody plants producing NTFPs in Northeast China are *Aralia elata* and *Eleutherococcus senticosus* (Wan et al., 2016; Jin et al., 2024b). *A. elata* is mostly found in the lower hinterland of mountains on fertile, well-drained semi-aerial and shady slopes, in neutral dark brown soils (Wei et al., 2019b; Yamawo and Tomlinson, 2023). It is characterized by low fruit set, poor seed dispersal and low germination, which makes its sexual reproduction difficult. *E. senticosus* prefers warm and humid climate, suitable for growing in moist and relatively fertile soil, often distributed in mixed coniferous and broad-leaved forests or broad-leaved forests. Its seeds have an innate dormant character and are less capable of natural regeneration under natural conditions in the wild without human intervention (Wang et al., 2019). *A. elata* is a protected plant of vulnerable level in the IUCN Red List of Threatened Species, and *E. senticosus* is an endangered natural medicinal plant under national key protection. Both economic forest trees are medicinal food homology plants in the family of Araliaceae and are widely popular. The related forest products of *A. elata* have a variety of chemical constituents such as terpenoids and flavonoids, which are widely used in the treatment of hepatitis and rheumatoid arthritis (Xu et al., 2022; Yin et al., 2024). The related forest products of *E. senticosus* have chemical components such as acanthopanax glycosides and lignans, which are widely used in the treatment of hypertension, rheumatism and ischemic heart disease, and have anti-fatigue, sleep-improving and memory-enhancing effects (Graczyk et al., 2021; Li et al., 2022).

However, climate change is having a huge impact on natural ecosystems and species survival around the world, and the magnitude of this impact is gradually increasing (Bellard et al., 2012; Mantyka-Pringle et al., 2012; Urban, 2015). Since the 1950s, the global average temperature has risen steadily and is expected to rise by 1.5-2.1°C by 2050. At the same time, changes in precipitation patterns will lead to more severe polarization in arid and humid regions (O'Neill et al., 2016; Naeem et al., 2019). Furthermore, land use changes caused by human activities have reduced the area of plant habitats and increased the risk of natural plant populations being threatened. The confluence of climate warming and intensified human interventions is progressively causing suitable habitats for numerous species to shift towards higher latitudes and elevations (Wani et al., 2023). In most areas, the resources of *A. elata* and *E. senticosus* are affected by these two factors, resulting in a serious threat to their natural populations and affecting the sustainable development of natural product resources (Zhang et al., 2021).

Understanding the distribution and important influencing factors of two economic trees is helpful for the protection and development of species. The species distribution model can predict the suitable habitat distribution of species under current and future conditions based on species distribution data and environmental data (Elith and Leathwick, 2009). The current popular species distribution models include generalized linear model (GLM), random forest (RF), Ecological Niche Factor Analysis (ENFA), Bioclimatic Prediction System (Bioclim), Genetic Algorithm for Rule-set Prediction (GRAP) and maximum entropy mode (MaxEnt). Among them, MaxEnt, as a machine learning method, is one of the most commonly used species distribution models. Compared with other models, MaxEnt has high simulation accuracy and is characterized by easy operation and short running time (Phillips et al., 2006). When the sample size of a species is limited, MaxEnt’s performance is usually better than other models (Morales et al., 2017). Therefore, it has become one of the effective models to study the distribution of species and is widely used in the simulation of suitable habitats for plants (Wei et al., 2018; Li et al., 2024).

Here, we use the MaxEnt model to simulate the potential suitable habitats of two economic forest trees in the present and future, and analyze the characteristics of suitable environmental factors of two economic forest trees. This study focuses on three key objectives: (1) Predicting the suitable habitats of *A. elata* and *E. senticosus* in current and future periods (2070s, 2090s); (2) Analyzing the relationship between main environmental factors and suitable habitats; (3) Determine the suitable habitat changes of *A. elata* and *E. senticosus* in the future. This study will provide a scientific basis for the sustainable development and utilization of understory economic trees in Northeast China.

## 2 Materials and methods

### 2.1 Study area and species data

**Figure 1:**
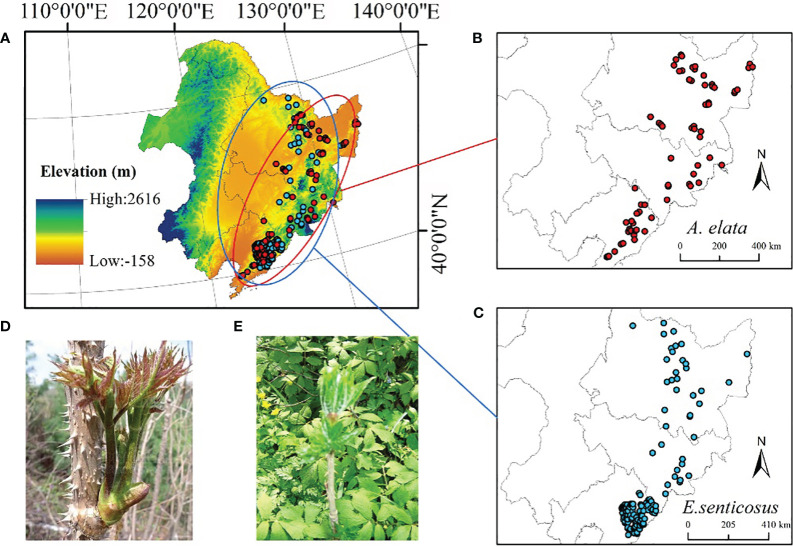
**(A)** Study area. **(B)** *A*. *elata* distribution point (n=70) **(C)** *E*. *senticosus* distribution point (n=124) **(D)** *A*. *elata* shoots (edible part) **(E)** *E*. *senticosus* leaves (edible part).

The northeastern region of China includes Heilongjiang, Jilin, Liaoning, and parts of neimenggu autonomous regions (**Figure 1A**). The Changbai Mountain region in the east has always been the richest in biodiversity in northern China and is important for regional biodiversity conservation.

In this study, we collected specimen records of two economic forest trees from publicly available databases, including the Global Biodiversity Information Facility (https://www.gbif.org), the Chinese Virtual Herbarium (https://www.cvh.ac.cn), the Teaching Specimen Resource Sharing Platform (https://mnh.scu.edu.cn). Publicly available species record data were also obtained from published literature (Guo et al., 2019; Wei et al., 2019a; Tian et al., 2021). We only obtained records of specimens with clear latitude and longitude information. A total of 61 records were collected to document the distribution of *A. elata* and 56 records were collected to document the distribution of *E. senticosus*. In addition, we conducted an extensive field survey of the distribution of the two economic forest trees in the Northeast region during 2021, collecting and recording a total of 90 distribution point data for *A. elata* and 83 distribution point data for *E. senticosus*.

A total of 151 A*. elata* distribution point data and 139 *E. senticosus* distribution point data were collected in both ways. The data tended to have high similarity due to data collection through multiple data sources. We removed duplicates and ensured the availability of each piece of data. Meanwhile, in order to reduce the spatial autocorrelation of the data and to ensure that the distance between any two points is greater than 1km, we used ArcGIS 10.4 for de-duplication and elimination of too close points. Finally, we used 70 A*. elata* distribution data and 124 *E. senticosus* distribution data were used to build the model. The distribution of the two economic forest trees is shown in **Figure 1**, and most of the points are distributed in Liaoning Province.

### 2.2 Climate data

Bioclimatic variables are important variables affecting changes in suitable habitats for species, and we selected 19 bioclimatic variables from the World Climate Database (https://www.Worldclim.org/) at a resolution of 1km (Fick and Hijmans, 2017). We choose the same variables for the current period and the future period. Two periods, 2070s (2060-2080) and 2090s (2080-2100), were selected for the future climate variables. Future climate data for the two periods were selected from the BCC-CSM2-MR model, which is capable of reasonably simulating regional climate change trends in China (Liu et al., 2022b). This model can reflect the global energy balance, accurately reflect the complexity of regional climate change in China, and have high simulation accuracy for factors such as atmospheric temperature and precipitation (Wu et al., 2021a). It has also been shown that the model may overestimate the complexity of climate change in some regions, but mainly in the western region (Jin et al., 2024a). The future climate data use the latest Coupled Model International Comparison Program 6 (CMIP6) Shared Socio-Economic Pathways and Typical Concentration Pathways Combined Scenarios (SSP) of SSP126 (low forcing scenario), SSP245 (medium forcing scenario), and SSP585 (high forcing scenario).

Species distribution models usually assume that land use/land cover (lulc) data are static to estimate future changes in species suitable habitat. However, in addition to climate change, future changes in lulc are one of the most important factors affecting changes in suitable habitat for species. Land use factors can interact with climate factors and the negative impacts of climate change can be altered through appropriate land management as an adaptation measure (Oliver and Morecroft, 2014; Mair et al., 2018; Seaborn et al., 2021). Here, we consider the effects of land use changes on suitable habitat. Current land use data from the Resources and Environment Data Center of the Chinese Academy of Sciences (http://www.resdc.cn). Future land use data is obtained from land use simulations using the Future Land Use Simulation (FLUS) model based on the Land Use Harmonization (LUH2) project (Liao et al., 2020). The land use data has a resolution of 1km and is divided into six land classes: 1: forest; 2: grassland; 3: unutilized land:4: cropland:5: urban:6: water. Future land use changes are similarly modeled land use data for the four SSP scenarios.

Additional data are necessary, although these are the same data that will change somewhat in the future. However, we make the assumption that some variables will not change in the future. This is because considering some variables as static variables as environmental variables can achieve better or not worse modeling results than ignoring them. This is especially important when dynamic variables interact with these static variables (Stanton et al., 2012).

The topographic data were used as 3 variable data of elevation, slope, and aspect. Slope and aspect were produced using ArcGIS 10.4 software based on the digital elevation model. Soil is an important factor in the survival of plants, and the use of soil factors often leads to better performance in species distribution models (Oliveira et al., 2021). Soil bulk, Soil clay, Soil organic carbon, Soil silt, and Soil pH were used for soil data in this study. Human footprint data from The Socioeconomic Data and Applications Center (http://sedac.ciesin.columbia.edu).

### 2.3 Data processing

Environmental variables tend to be highly correlated with each other, which can influence the model to produce misleading interpretations (Liu et al., 2022a; Li et al., 2023). Therefore, we utilized the SDX toolbox2.5 multicollinearity test to screen for variables that have some correlation with each other (Brown et al., 2017). When the absolute value of the Pearson correlation coefficient between any 2 environmental variables was greater than or equal to 0.8, the 2 variables were compared in terms of their contribution in the MaxEnt model, and the environmental variable with the higher contribution was retained. We finally selected 15 environmental variables for modeling from 28 variables (**Table 1**).

**Table 1:** Environmental factors used in the model and their contributions.

Variables	Description	*A.elata*		*E.senticosus*	
		Percent contribution	Permutation importance	Percent contribution	Permutation importance
Bio1	Annual mean temperature (°C)	13.2	13.6	2.3	2.5
Bio2	Mean diurnal range (°C)	9.4	14.5	3.6	12.9
Bio3	Isothermally	1.4	5.4	2.7	5.6
Bio12	Annual precipitation (mm)	27.4	21.8	61.7	44.8
Bio15	Precipitation Seasonality	15.3	13.6	8.2	6.5
Lulc	There are 6 types: (1) Forest; (2) Grassland; (3) Barren; (4) Cropland; (5) Urban; (6) Water	16	4.9	10.2	4.4
Dem		4.1	5.1	0.7	2.4
Slope	Slope (°)	5.6	6.7	4.8	11.9
Aspect		1.8	2.4	1.3	1.8
T_bulk	Soil bulk(kg/dm^3^)	0.1	0.5	0.3	0
T_clay	Soil clay(%)	0.9	3.2	0.5	1.1
T_oc	Soil organic carbon(%)	1.1	1.5	0.1	0.2
T_ph	Soil pH	0.3	0.7	0.1	0.1
T_silt	Soil silt(%)	1.4	2	1.7	2.8
Hf	Human footprint	2	4.2	1.8	3

### 2.4 Modeling methods and analysis

We performed modeling and analysis based on species distribution data and screened environmental data. In the MaxEnt model, we utilize 25% distribution data as the test set for validation and 75% distribution data as the training set to build the model. The number of iterations is set to 5000, and the model is set to repeat 10 times to ensure the randomness of the selection of the training set for the test set (Yi et al., 2016), and uses Bootstrap as the operation type, leaving the other parameters as defaults (Phillips and Dudík, 2008). We assessed the accuracy of the model predictions using AUC values between 0 and 1. The closer the AUC value is to 1, the better the model performs. It is generally accepted that an AUC greater than 0.9 is sufficient for the model predictions to be used. There are many ways to classify suitable habitat indices, among which threshold classification using maximizing the sum of sensitivity and specificity (max SSS) is the most scientific (Liu et al., 2013, Liu et al., 2016). Our predictions used max SSS to categorize the study area into suitable and unsuitable habitats, and then categorized suitable habitats into three suitability classes, low, medium, and high, using the natural discontinuity point method. Meanwhile, in order to clarify the trend of changes in the spatial pattern of suitable habitat under current and future climate scenarios, the Distribution changes between binary tool in SDMtoolbox2.5 was used to calculate the spatial changes of suitable habitat, and the Centroid changes tool was used to calculate the spatial distribution center of mass of suitable habitat. The migration of the center of mass of the spatial distribution of suitable habitat was calculated using the Centroid changes tool.

## 3 Results

### 3.1 Model performance optimization and calculation results

**Figure 2:**
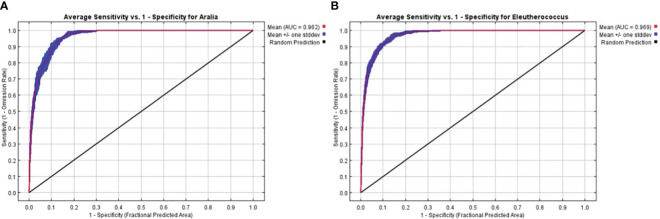
AUC values of the model in the current period **(A)** *A. elata* **(B)** *E*. *senticosus*.

We assessed the accuracy of the MaxEnt model using AUC values (**Figure 2**). The AUC values for all replicates in the MaxEnt build process were greater than 0.9. The average AUC values for the current period model runs for *A. elata* and *E. senticosus* were 0.962 and 0.969. This indicates that MaxEnt performs well and has high reliability.

### 3.2 Main environmental variables and response curves

**Figure 3:**
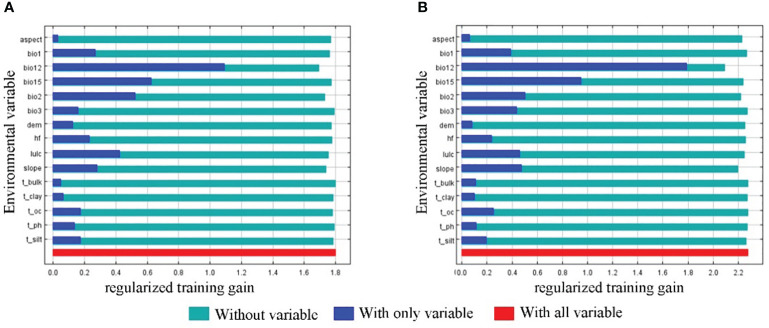
Jackknife test of the environmental variables **(A)** *A.elata* **(B)** *E.senticosus*.

**Figure 4:**
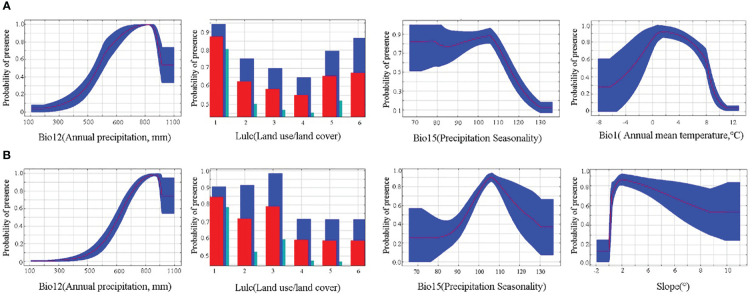
Response curves for major environmental variables. **(A)** *A*. *elata* **(B)** *E*. *senticosus*.

The distribution of the two economic forest trees was influenced to varying degrees by the 15 environmental variables (**Figure 3**). The top 4 contributing factors to the potential geographic distribution of *A. elata* were Annual precipitation (27.4), land use (16), Precipitation Seasonality (15.3), and Annual mean temperature (13.2), with a cumulative contribution of 71.9%. The top 4 factors with importance values are Annual precipitation (21.8), Mean diurnal range (14.5), Precipitation Seasonality (13.6), Annual mean temperature (13.6) with a cumulative importance value of 70.2. Univariate response curves were plotted using MaxEnt (presence probability: *p*≥0.6) (**Figure 4A**). The results showed that the presence probability of *A. elata* was maximum when the Annual precipitation was 921 mm. The presence probability of *A. elata* was maximum when the land use type was forest. When Precipitation Seasonality is less than 113.8, it is the best survival range for *A. elata*. The presence probability of *A. elata* was greatest when the Annual mean temperature was 1.6°C.

The top 4 contributing factors to the potential geographic distribution of *E. senticosus* were Annual precipitation (65.6), land use (8.2), Precipitation Seasonality (7), and slope (5) with a cumulative contribution of 85.8%. In terms of importance, the top 4 environmental factors were Annual precipitation (44.1), Mean diurnal range (15.2), slope (12.9), and Precipitation Seasonality (5.9) with a cumulative importance of 78.1%. The presence probability of *E. senticosus* was maximum when Annual precipitation was 965.9 mm. The presence probability of *E. senticosus* is maximum when the land use type is forest. The presence probability of *E. senticosus* is maximum when Precipitation Seasonality is 106.2. The presence probability of *E. senticosus* is maximum when Slope is 2° (**Figure 4B**). Annual precipitation had a dominant effect on both economic forest trees, and Annual precipitation had information not present in the other variables and the most useful information.

### 3.3 Current suitable habitat

**Figure 5:**
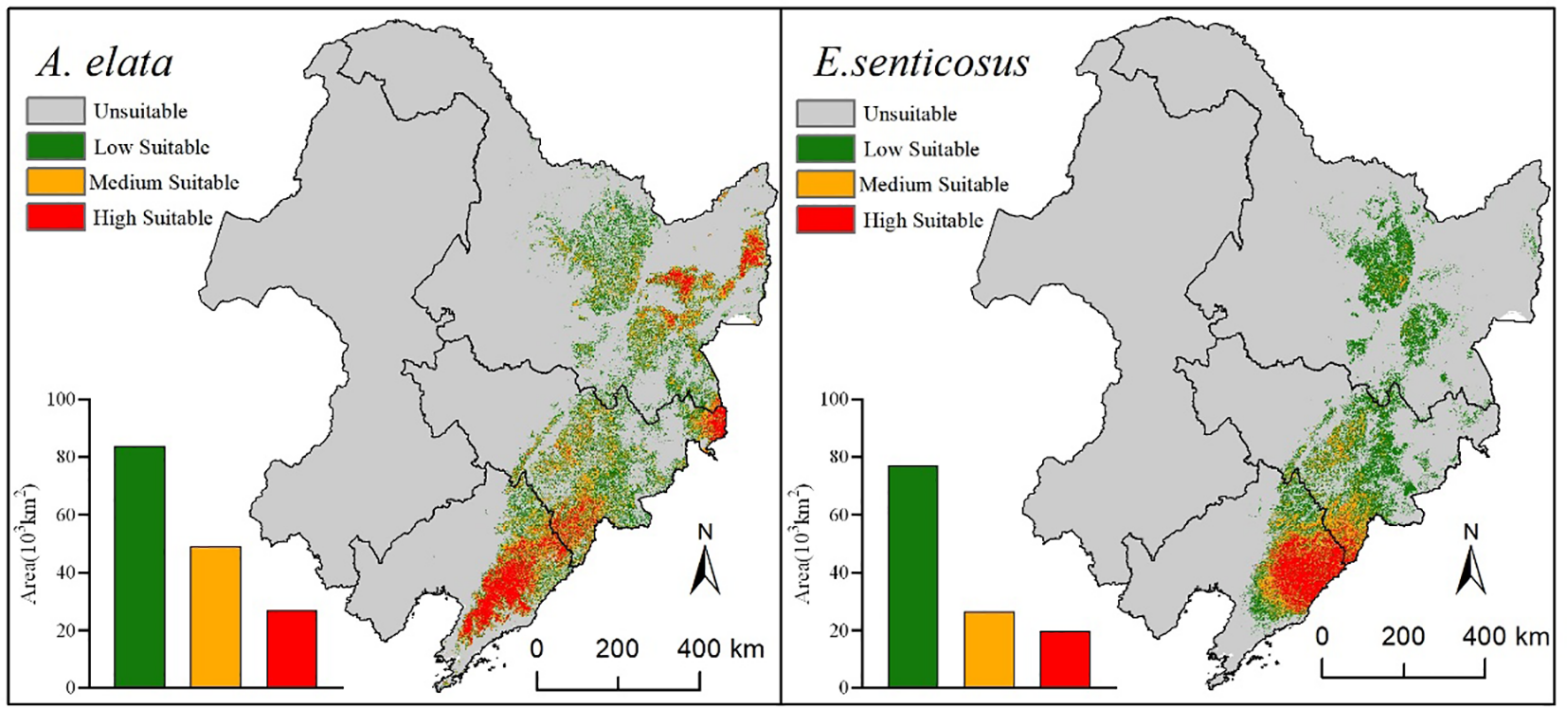
Current suitable habitat and area for *A. elata* and *E. senticosus*.

Based on the predictions of the model, the suitable habitats of *A. elata* and *E. senticosus* were categorized into four types of highly suitable habitats, moderately suitable habitats, and low and unsuitable habitats (**Figure 5**). The highly suitable habitat of *A. elata* has an area of 27,082 km^2^, accounting for 16.93% of the total area. The medium suitable habitat was distributed around the high suitable habitat, with an area of 49,109 km^2^, accounting for 30.70% of the total suitable habitat area. The area of low suitable habitat is 83,759 km^2^, accounting for 52.37% of the total suitable habitat area. The suitable habitat area of *E. senticosus* was smaller than that of *A. elata*, with a total area of 123,449 km^2^. 19,789 km^2^, or 16.03% of the total suitable habitat area, was highly suitable habitat. The medium suitable planting area is 26,516 km^2^, accounting for 21.48% of the total suitable habitat. The area of low suitable habitat is 71,756 km^2^, accounting for 62.49% of the total suitable habitat. Overall, suitable habitat for both *A. elata* and *E. senticosus* was found in Liaoning, Jilin and Heilongjiang provinces, but the area of highly suitable habitat for both trees was highest in Liaoning province, especially in the Changbai Mountain Residue area. The total suitable habitat area of *E. senticosus* was smaller than that of *A. elata*, and the proportion of low suitable habitat area was higher among the suitable habitats of *E. senticosus*. The high suitable habitats of the two trees are mainly located in the eastern part of Liaoning Province, and the overlap area is large.

### 3.4 Future suitable habitats

**Figure 6:**
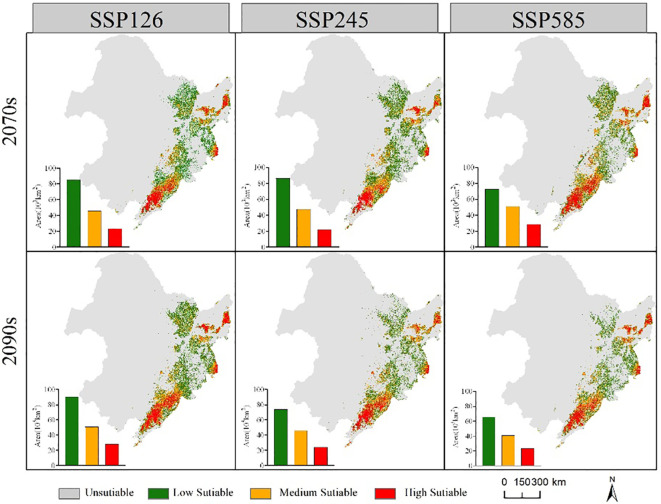
Range and area of suitable habitat for *A. elata* under different future climate scenarios.

**Figure 7:**
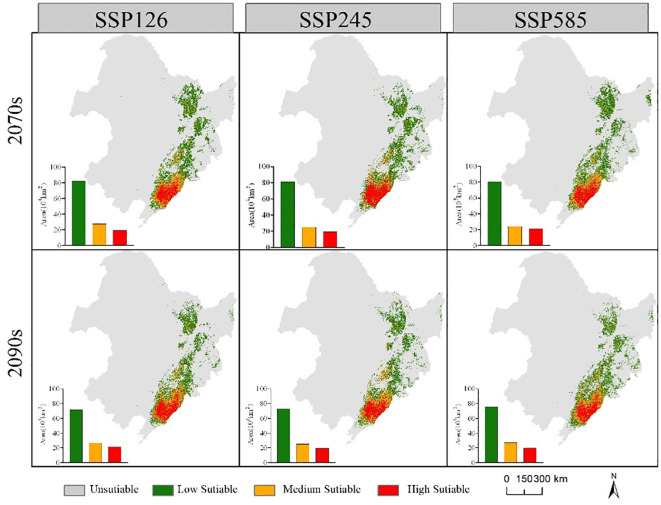
Range and area of suitable habitat for *E. senticosus* under different future climate scenarios.

The suitable habitat range of the two economic forest tree species in the future is similar to the current period (**Figures 6**, **7**). In the future, the suitable habitats of the two trees will still be mainly located in the eastern part of the Northeast, and most of the areas will overlap. Compared with the current suitable habitat, the total suitable habitat area of *A. elata* decreased under different climate scenarios in 2070s. In the SSP126 scenario of 2090s, the suitable habitat area of *A. elata* increased. Compared to the current suitable habitat, *E. senticosus* increased in total suitable habitat area under different climate scenarios in the 2070s and decreased in total suitable habitat area in the 2090s. Under most climate scenarios of 2090s, the suitable habitat area of the two trees showed a decreasing trend.

**Figure 8:**
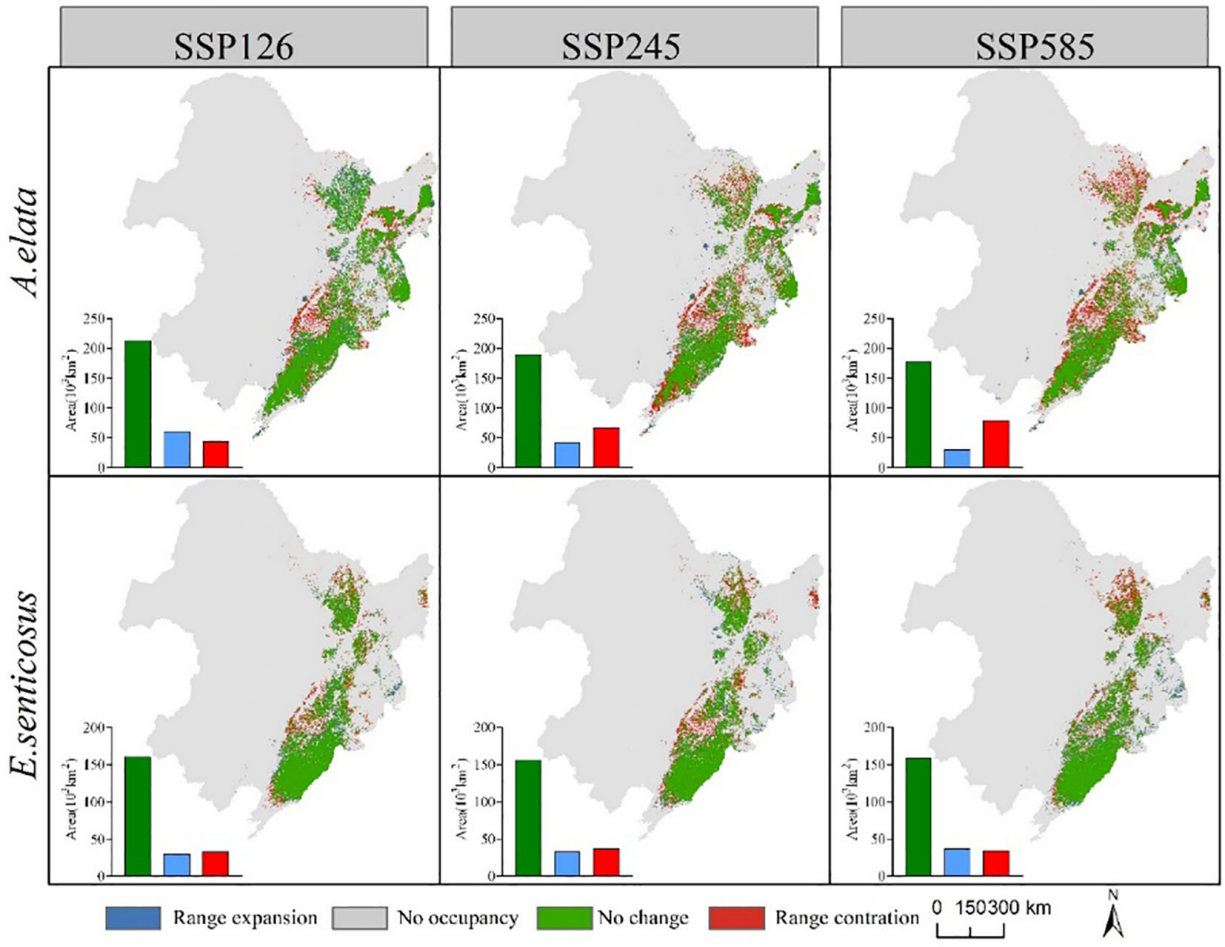
Changes in the area of suitable habitat for *A. elata* and *E.senticosus* under different scenarios in 2090s.

**Figure 8** shows the spatial distribution of changes in suitable habitat area for the two economic forest trees under three climate scenarios in the 2090s. The area of suitable habitat for *A. elata* loss increased gradually with increasing climatic stress. The area of suitable habitat lost under the three climate scenarios SSP126, SSP245, and SSP585 was 43,878, 67,534, and 79.532 km^2^, respectively. At the same time, the area of suitable habitat for expansion gradually decreased. The areas of expansion under the three climate scenarios SSP126, SSP245 and SSP585 are 61,391, 42,693 and 30,450 km^2^, respectively. There was no significant difference in the suitable habitat area for *E. senticosus* expansion or contraction. The contracted areas were mainly located at the periphery of the current suitable habitat, and the expanded areas were located in the eastern part of Jilin Province. The contraction of suitable habitat under the three climate scenarios SSP126, SSP245, and SSP585 was 32782, 37188, and 34792 km^2^, respectively, and the expansion of suitable habitat was 30669, 32819, and 36686 km^2^, respectively.

**Figure 9:**
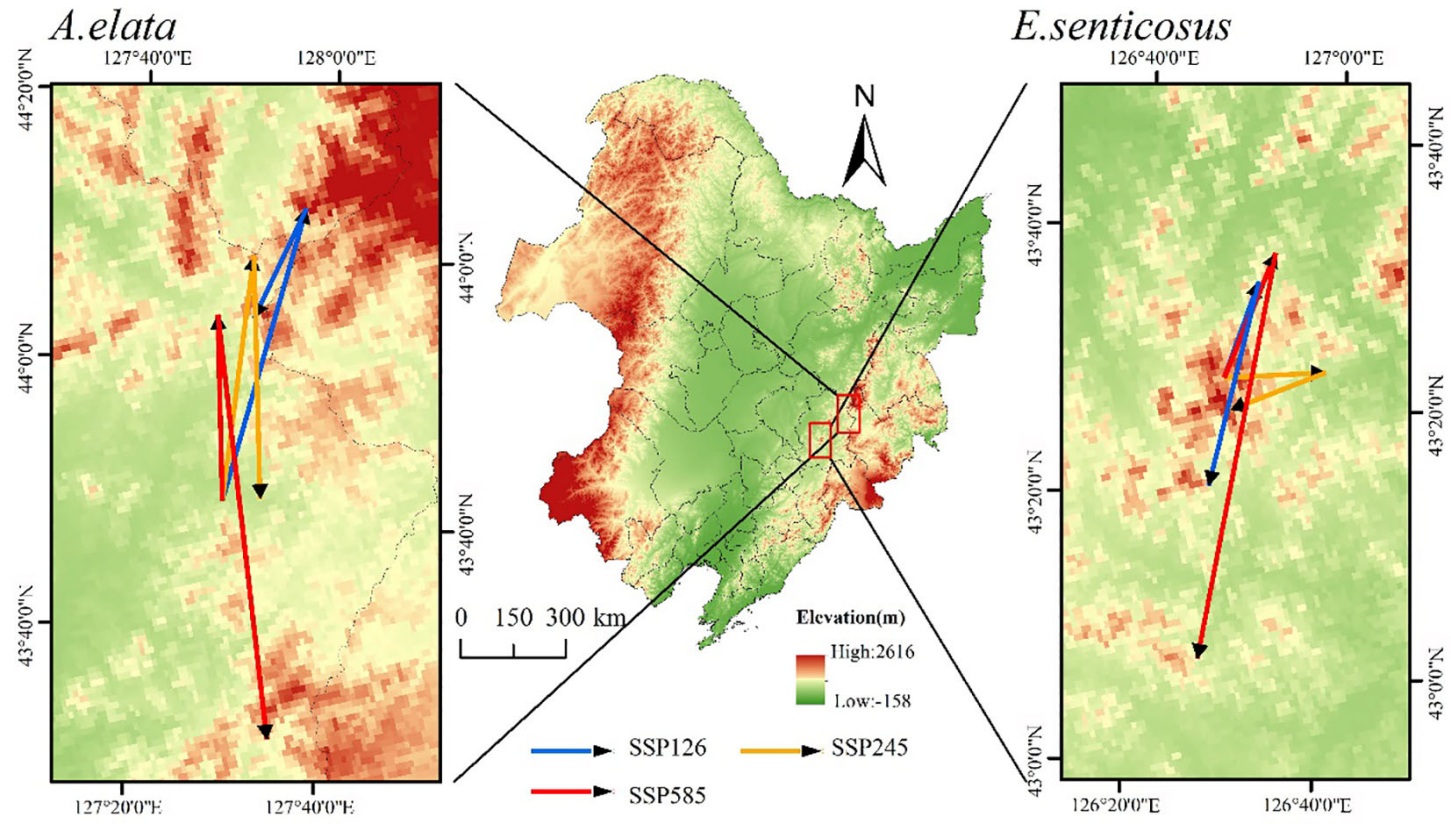
Changes in the centroid of suitable habitat for *A. elata* and *E. senticosus* under different scenarios.

The future migration of the suitable habitat centroids of the two trees is shown in **Figure 9**. Finally, in the SSP126 climate scenario, the centroid of *A. elata’s* suitable habitat migrated northward, while in other climate scenarios it migrated southward. Under the two scenarios of SSP126 and SSP 585, *E. senticosus* showed a migration route from north to south, and a migration route from east to west under the SSP245 scenario.

## 4 Discussion

### 4.1 Restriction of environmental variables on geographical distribution

In this study, the MaxEnt model was used to explore the extent of suitable habitat and environmental factors affecting the distribution of *A. elata* and *E. senticosus* under climate change and land use change. Species distribution is the result of long-term adaptation between species and the natural environment, which includes abiotic factors such as climatic, topographic and soil factors, as well as biotic factors such as human activities and land-use change (Qin et al., 2017; Yan et al., 2020). Among them, factors such as topography and soil change little with time, and climate factors and land use factors often change greatly. Annual precipitation (bio12) is the most important climatic variable affecting both plant species, and previous studies on *A. elata* have shown that soil moisture can affect the photosynthetic properties of leaves and thus plant growth (Zhao et al., 2018). Specifically, both economic forest trees require more suitable precipitation to survive. Not only that, precipitation seasonality (bio15) is also crucial. However, the coefficient of variation of precipitation seasonality for suitable *E. senticosus* was greater than that for *A. elata*, suggesting that *E. senticosus* requires more water in summer. In addition, survival of *A. elata* was affected by Annual mean temperature (bio1) and survival of *E. senticosus* was affected by slope. This suggests that although precipitation plays a decisive role in the potential distribution of the two economic forest trees, topography and temperature also play a role to some extent. These factors may interact with each other to influence the potential distribution pattern of species (Wang et al., 2023).

Climate change factors tend to have a more significant impact on biodiversity than land use change (Barras et al., 2021; Preau et al., 2022). In this study, forests was the most suitable land type for both trees. Compared to climate factors, land use factors have a smaller magnitude of influence on both plants. However, the role it plays cannot be ignored as climate change may interact with land use. This effect may be positive or negative, but it opens up the possibility of utilizing the interaction of climate change and land use change to reduce the negative impacts of climate change (Zhao et al., 2023). Previous research on *Davidia involucrata* suggests that future patterns of land use will offset the negative impacts of climate change on it (Tang and Zhao, 2022).

### 4.2 Changes in future suitable habitats

According to the distribution of suitable habitats, the high suitable habitats of *E. senticosus* are very concentrated, mainly distributed in Liaoning Province. The high suitable habitat of *A. elata* is distributed in all three provinces, and the area from large to small is Liaoning Province, Jilin Province and Heilongjiang Province. The high suitable habitat of the two trees has a large overlap area, which is located in the eastern part of Liaoning Province. This is consistent with the actual situation of the non-wood forest product industry in the eastern part of Liaoning Province, *A. elata* and *E. senticosus* have a large area of distribution (Zhang et al., 2021). In different climate scenarios in the future, the suitable habitat of *A. elata* showed high sensitivity. With the increase of greenhouse gas emissions, the area of expansion area is gradually smaller than that of contraction area. The area of suitable habitat shrinkage and expansion of *E. senticosus* changed little in different scenarios. This may be due to the impact of future climate change, the seasonal difference of annual precipitation increases, and the annual average temperature increases (Wang et al., 2023; Zhang et al., 2024). *A. elata* was greatly affected by seasonal precipitation, and *E. senticosus* was less affected by seasonal precipitation. In addition, the annual average temperature is also one of the main factors affecting the distribution of *A. elata* (**Figure 3**).

From the perspective of centroid migration, the centroid migration trends of the two trees are generally similar, showing a migration route from north to south. With the increase of climate warming, the overall migration distance of the suitable habitats of the two trees is increasing, and the migration distance is the largest in the SSP585 scenario. This may be due to the migration of plants to higher altitudes and fragmentation of suitable habitats as a result of future climate and land use changes (Tian et al., 2023; Wang et al., 2023). The synchronous migration of the two trees indicates that this is the same way these species survive in response to climate change.

### 4.3 Countermeasures for the conservation and utilization

Previous studies have shown that global warming can lead to the expansion, displacement, or contraction of suitable habitats for species, thus affecting their geographic distribution (Gebrewahid et al., 2020; Wu et al., 2021b). Our projections suggest that the area of suitable habitat for *A. elata* will increase under the ssp126 scenario in 2090s. In addition to this, the area of suitable habitat for both *A. elata* and *E. senticosus* will shrink in 2090s. Based on the above findings, we propose the following recommendations for the nature conservation and sustainable development and utilization of *A. elata* and *E. senticosus* in the Northeast.

(1) Implement targeted protection management measures in different regions. First, suitable habitats stabilized by the two economic forest trees were identified as priority conservation areas. Such areas are not seriously affected by climate change and can serve as priority areas for species reintroduction or species return. Precipitation factors should be considered first when selecting areas for species regression. It has been shown that drought stress inhibits photosynthesis and growth of *E. senticosus* (Xu et al., 2019). Because annual precipitation is an important environmental factor influencing the distribution of the two economic forest trees and because the reintroduction of *E. senticosus* should be carried out taking into account the effect of slope. Secondly, identify the areas where suitable habitats will be lost in the future as key areas of concern. The protection department should conduct inspections of individuals in such areas to comprehensively understand the actual distribution and population status of *A.elata* and *E.senticosus* in high-risk areas. For individuals with poor growth, certain protective measures should be taken, and relocation protection can be carried out if necessary. In the future, in the development and utilization of natural population resources, a certain logging intensity should be controlled to achieve the goal of sustainable development (Zhang et al., 2021). Prohibit private logging that does not follow a sustainable use plan in order to mitigate the negative impacts of human activities on natural populations.

(2) Mobilizing local residents to establish forest economy industries. Artificial cultivation is a key method for the sustainable development of non-timber forest products. The use of cultivation techniques can enhance local economic development and thus reduce the intensity of over-harvesting of natural populations. In order to better promote the establishment of an industrial system, it is necessary to use controlled experiments to study the environmental factors affecting the cultivation process and to explore mature cultivation models. It has been shown that the semi-fertilized state is the best way to ameliorate the adverse effects of light on *A. elata* seedlings and to improve their survival rate (Zhang et al., 2022). Interplanting of *Larix gmellini* with *A. elata* not only enhances economic benefits, but also improves soil chemistry and microbiological properties in larch plantations (Gao et al., 2022). Composite systems of *Pinus koraiensis* and *E. senticosus* are equally economically valuable and easy to implement with viable combined conservation measures (Wan et al., 2016). In addition, in areas unsuitable for *A. elata* and *E. senticosus*, artificial facilities can be utilized to regulate the temperature, moisture, soil, and other factors of the planting environment in order to achieve environmental conditions suitable for their growth. However, the planting of artificial forests will affect the succession and natural regeneration of natural vegetation and forests. We should find a balance between economic development and sustainable development of ecological environment. Therefore, the local development of under-forest economy does not mean that the area of economic forest will be greatly expanded.

### 4.4 Potential limitations

The geographical distribution of species is the result of the interaction of multiple biotic and abiotic factors, and the interplay between these factors is complex. Interactions between environmental factors may present opportunities to address climate change, but the MaxEnt model utilized in this study only focused on the effects of a single environmental factor on species. Future research could focus on the effects of interactions between environmental factors on species distributions and explore how they can be utilized to reduce the negative impacts of climate change. In addition, our study did not focus on current and future positive impacts of nature reserves or other artificial conservation measures on the two economic forest trees, and also ignored domestication changes made by the species in response to future environmental changes. This may overestimate the area of suitable habitat shrinkage for both plants. Therefore, more in-depth studies are needed in the future to incorporate future positive factors into the modeling of suitable habitat for the species.

## 5 Conclusion

*A. elata* and *E. senticosus* are important understory economic tree species, but their natural resources are severely damaged. In this study, we used the MaxEnt model to simulate the spatial distribution and area changes of suitable habitats for *A. elata* and *E. senticosus*, and explored measures for the development of sustainable use of non-timber forest products. It was found that the suitable habitats for both economic forest trees were mainly located in the eastern Changbai Mountain area. Annual precipitation was the main factor affecting the two economic forest trees. We predict that under future climate scenarios, *A. elata* suitable habitat area is more likely to decrease, and *E. senticosus* suitable habitat area will decrease. Both economic forest trees require active conservation management and rational exploitation strategies to halt the widespread loss of their habitats and to promote the sustainable exploitation of non-timber forest products.

## Data availability statement

The raw data supporting the conclusions of this article will be made available by the authors, without undue reservation.

## Author contributions

XL: Methodology, Writing – original draft, Data curation. BC: Conceptualization, Writing – review & editing, Supervision, Visualization. YH: Funding acquisition, Investigation, Writing – review & editing, Conceptualization, Visualization. XJ: Funding acquisition, Writing – review & editing, Project administration, Supervision, Visualization.
